# Supplementary material for: Machine learning electronic structure methods based on the one-electron reduced density matrix
Source: Nat Commun. 2023 Oct 7;14:6281. doi: 10.1038/s41467-023-41953-9 (PMC10560258; doi:10.1038/s41467-023-41953-9)
Supplement: Supplementary file 1 — Supplementary information [file 41467_2023_41953_MOESM1_ESM.pdf]

Supplementary information for:

Machine Learning Electronic Structure Methods Based On The One-Electron Reduced Density Matrix

Xuecheng Shao, Lukas Paetow, Mark E. Tuckerman, and Michele Pavanello

QMLearn Software

The QMLearn software is available on GitLab at `https://gitlab.com/pavanello-research-group/qmlearn`. The version used for this work is 0.0.1.

Specific QMLearn dependencies and their version used for all calculations are listed as follows:

- ase (3.22.1)
- h5py (3.7.0)
- numpy (1.23.1)
- pyscf (2.0.1)
- scikit-learn (1.1.1)
- scipy (1.8.1)

Supplementary Figures

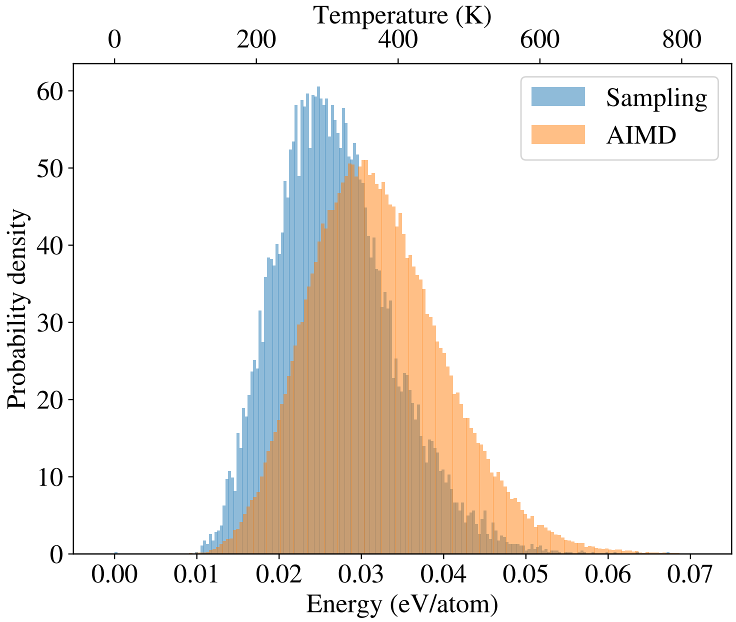

FIG. S1: Energy distribution of the training set compared to a dynamics trajectory. Comparison the energy distribution of an ab-initio molecular dynamics (AIMD) trajectory in the canonical ensemble, with $T = 300K$ and the distribution recovered from the sampling (see main text for details) for the benzene molecule. AIMD uses 138,000 steps. Our sampling method uses 13,824 structures.

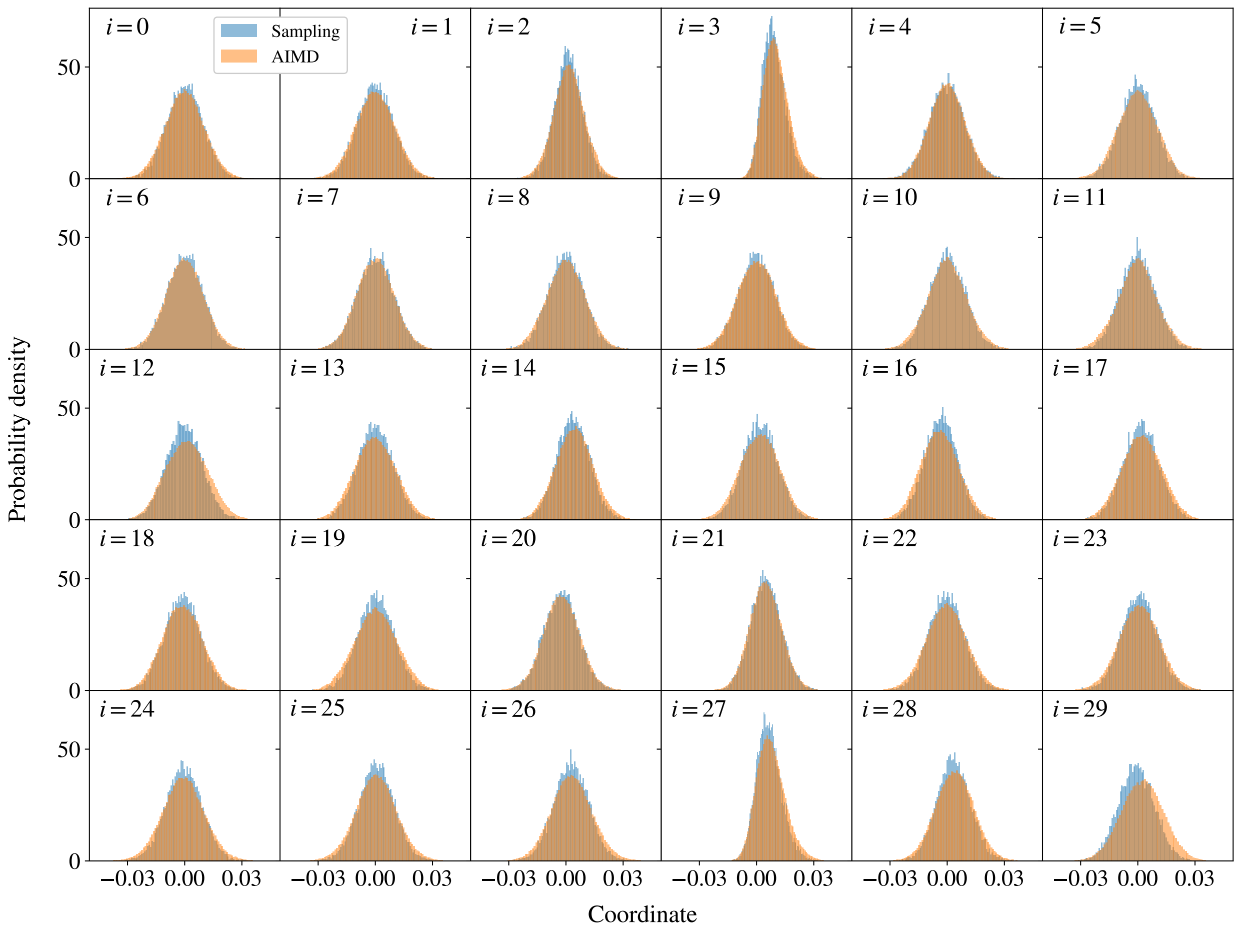

FIG. S2: Displacement away from the equilibrium geometry. Distributions of geometry displacements along each normal mode for an ab-initio molecular dynamics (AIMD) trajectory in the canonical ensemble at $T = 300K$ and the geometries found with the sampling method for the benzene molecule. The AIMD uses 138,000 structures. Our sampling method uses 13,824 structures for this molecule. The x-axis units are in energy-scaled coordinates ($x = \delta_i \cdot \Omega_i$, x being the x-axis value, δ_i the geometry displacement along the normal-mode coordinate, and Ω_i is the vibrational frequency associated with the normal mode).

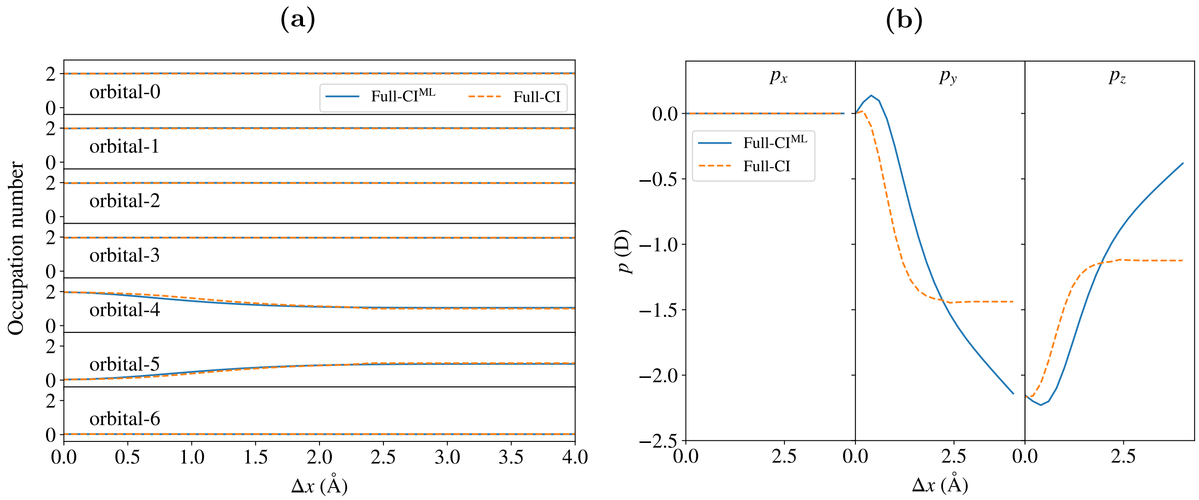

FIG. S3: Occupation numbers and dipole moment along a single-bond stretching coordinate. The occupation numbers, (a), and dipole moment components, (b), computed from full configuration interaction (Full-CI) and the surrogate model Full-CI^{ML} for the single bond stretching ($H_2O \rightarrow OH + H$) of H_2O.

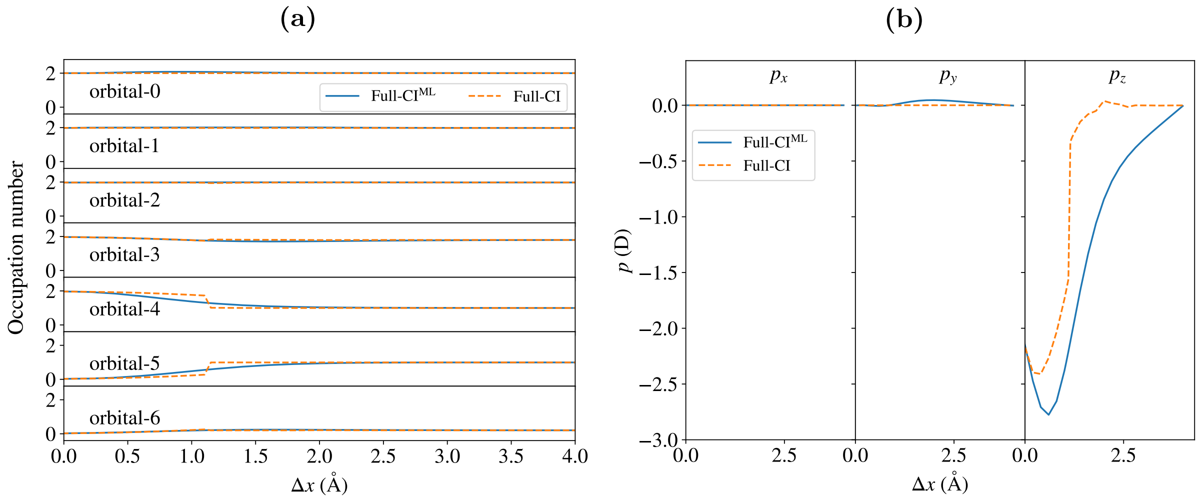

FIG. S4: Occupation numbers and dipole moment along a two-bond stretching coordinate. The occupation numbers, (a), and dipole moment components, (b), computed from full configuration interaction (Full-CI) and the surrogate model Full-CI^{ML} for the bond stretching coordinate breaking two OH bonds simultaneously ($H_2O \rightarrow O + H_2$) of H_2O.

(a)

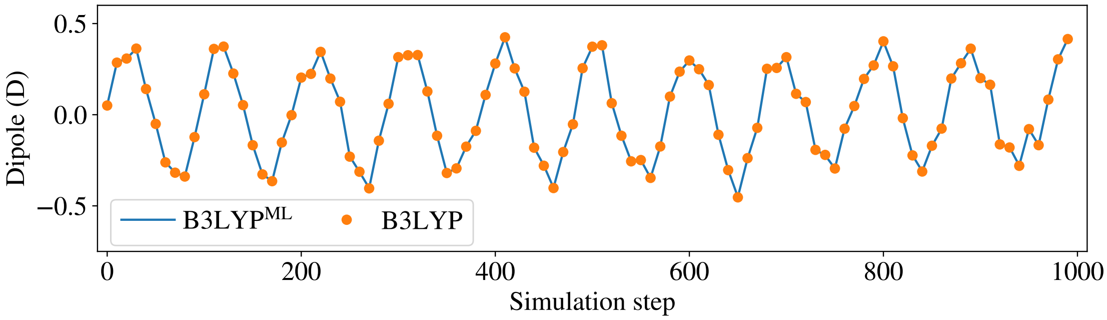

(b)

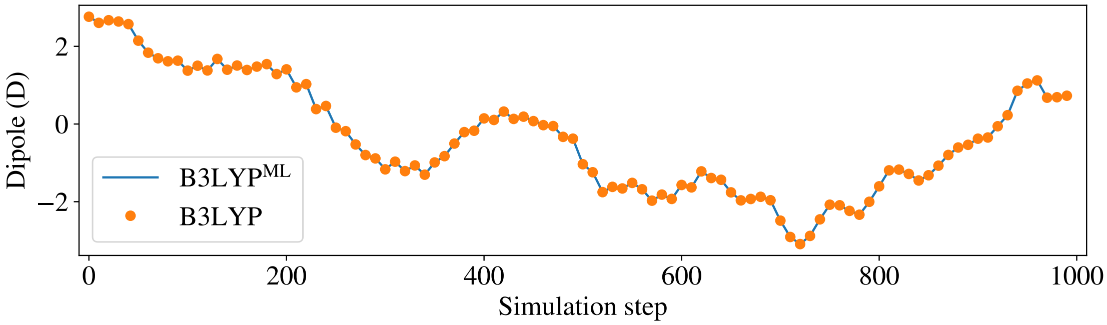

(c)

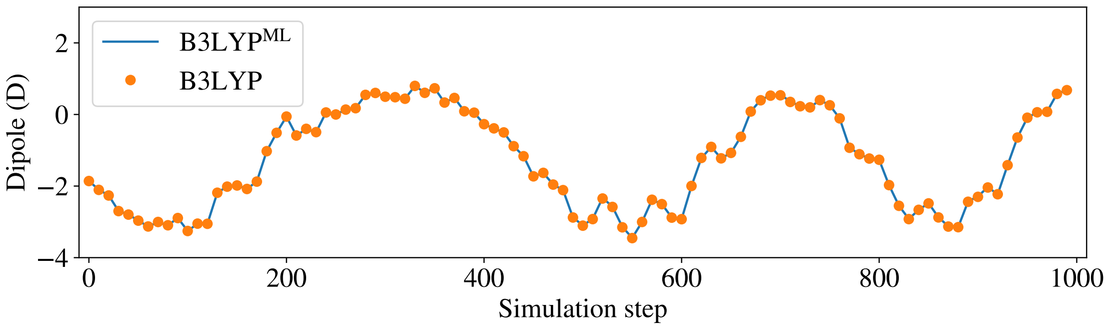

FIG. S5: Dipole moment along a dynamics trajectory. Magnitude of dipole moment vector along a 1000 steps ab-initio molecular dynamics trajectory in the microcanonical ensemble sampled every 10 steps of (a) benzene, (b) 1-propanol and (c) 2-propanol. The B3LYP^{ML} surrogate model values in blue lines, and the hybrid functional B3LYP values in orange dots.

Supplementary Tables

TABLE S1: Root mean square deviations (RMSDs) for all individual terms in the energy functional for the surrogate model LDA^{ML}. The RMSDs of Hartree-Fock exact exchange from the local density approximation (LDA) and surrogate LDA^{ML} 1-rdms are also shown in the table. Errors are given in kcal·mol^{-1}.

System		H_2O	CO_2	NH_3	CH_3OH	C_6H_6	1-propanol	2-propanol
Total	$LDA^{ML}[\gamma^p]$	0.0004	0.0020	0.0008	0.0050	0.0145	0.1640	0.0636
	$LDA^{ML}[\gamma^p + \Delta\gamma^p]$	0.0003	0.0020	0.0008	0.0014	0.0055	0.0270	0.0076
External	$LDA^{ML}[\gamma^p]$	1.1036	1.0953	0.9312	2.9543	2.8260	11.9038	10.5202
	$LDA^{ML}[\gamma^p + \Delta\gamma^p]$	0.1907	0.0710	0.0553	0.3523	0.8108	3.1885	2.5417
Kinetic	$LDA^{ML}[\gamma^p]$	0.2785	0.6084	0.5594	1.4603	1.1200	5.4203	4.1356
	$LDA^{ML}[\gamma^p + \Delta\gamma^p]$	0.1082	0.0513	0.0342	0.1909	0.3542	1.0903	0.9955
Coulomb	$LDA^{ML}[\gamma^p]$	0.9543	0.5808	0.4743	1.7104	1.9336	7.5814	7.2867
	$LDA^{ML}[\gamma^p + \Delta\gamma^p]$	0.0938	0.0275	0.0278	0.2131	0.5361	2.4095	1.7516
Exchange-Correlation	$LDA^{ML}[\gamma^p]$	0.0944	0.0759	0.0670	0.1981	0.1771	0.7921	0.6932
	$LDA^{ML}[\gamma^p + \Delta\gamma^p]$	0.0093	0.0051	0.0037	0.0266	0.0595	0.1806	0.1577
Exact exchange	$HF[\gamma^p]$	0.0858	0.0977	0.0663	0.2009	0.1557	0.8288	0.7236
	$HF[\gamma^p + \Delta\gamma^p]$	0.0092	0.0053	0.0035	0.0315	0.0519	0.1819	0.1557

TABLE S2: Root mean square deviations (RMSDs) for the occupation numbers of unpurified 1-rdms γ^p and $\gamma^p + \Delta\gamma^p$. All molecules are predicted by LDA^{ML}, except H_2O(Full-CI) which is predicted by full configuration interaction (Full-CI).

System		H_2O	H_2O(Full-CI)	CO_2	NH_3	CH_3OH	C_6H_6	1-propanol	2-propanol
# electrons		10	10	22	10	18	42	34	34
Occupations	γ^p	1.38E-04	3.66E-04	1.18E-04	1.31E-04	5.61E-04	1.31E-03	4.42E-03	3.33E-03
	$\gamma^p + \Delta\gamma^p$	1.80E-05	1.09E-05	2.89E-05	1.75E-05	3.84E-04	3.80E-04	3.66E-03	1.83E-03
